# Host Plant-Associated Wing Shape Variation of Pre-Dispersal Seed Predator Fruit Fly (Family Tephritidae) Based on Geometric Morphometrics

**DOI:** 10.3390/insects17060552

**Published:** 2026-05-27

**Authors:** Lixuan Kou, Fan Cheng, Mengke Liu, Di Zhang, Hao Wang, Zimeng Guan, Peisong Liu, Gang Wang

**Affiliations:** 1 Henan Key Laboratory of Germplasm Innovation and Utilization of Eco-Economic Woody Plant, Pingdingshan University, Pingdingshan 467000, China; koulx2015@163.com (L.K.);; 2 College of Geography and Environmental Engineering, Pingdingshan University, Pingdingshan 467000, China; 3 College of Chemistry and Chemical Engineering, Pingdingshan University, Pingdingshan 467000, China; 4 Henan Academy of Innovations in Medical Science, Institute of Electrophysiology, Zhengzhou 451100, China

**Keywords:** *Tephritis* sp. cf. *femoralis*, morphological differentiation, morphometrics, wing size and shape, host-associated morphological groups, alpine meadow

## Abstract

In plant–pre-dispersal seed predator interactions (e.g., flowerheads–flies), insect morphological differentiation is frequently driven by divergent host plants. However, wing morphological variation among flies associated with different flowerhead species has seldom been explored in natural alpine systems. In an alpine meadow of the Qinghai–Tibet Plateau, larvae of four host-associated morphological groups of *Tephritis* sp. cf. *femoralis* (1, 2, 3, 4) feed on four asteraceous species: *Saussurea nigrescens* (SN), *Carduus nutans* (CN), *Leontopodium leontopodioides* (LL), and *Anaphalis lactea* (AL). We examined the wing size and shape of these four groups using a landmark-based geometric morphometric approach. The host plants vary significantly in capitular diameter, dry weight, and depth. Within each host-associated group, female wing sizes (length, width and centroid size) were significantly larger than those of males. Wing size differed significantly among the four female and four male groups across host plants. Female ovipositor length varied in parallel with capitulum depth across the four host plants. Significant intersexual differences in wing shape were observed in SN, LL, and AL. Wing shape also varied distinctly among groups in both sexes across host plant capitula, enabling clear discrimination of host-associated groups. Linear discriminant analysis achieved high correct classification rates for both sexes, supporting clear morphological separation among host-associated groups. In conclusion, variations in wing size, wing shape and ovipositor length were associated with host traits. Overall, our study illustrates morphological differentiation correlated with host plants in flowerhead–fly pre-dispersal seed predator interactions in the alpine meadow system.

## Simple Summary

In an alpine meadow in the Qinghai–Tibet Plateau, larvae of four host-associated morphological groups of the pre-dispersal seed predator *Tephritis* sp. cf. *femoralis* (designated Tcf1, Tcf2, Tcf3, and Tcf4) feed on the flowerheads of four asteraceous species: *Saussurea nigrescens*, *Carduus nutans*, *Leontopodium leontopodioides*, and *Anaphalis lactea*. We examined the variations in wing size, shape, and ovipositor length of these fly groups in relation to these four host plant traits. Females had significantly larger wings than males in terms of length, width, and centroid size within each host-associated species. Wing size and shape differed significantly between sexes in each flowerhead based on geometric morphometrics. Wing shape also showed distinct differences among female and male groups from different host plants. Female ovipositor length was positively correlated with flowerhead depth. These morphological variations were closely aligned with host-specific traits. Our findings illustrate that host-associated insect trait variations (e.g., wing or ovipositor morphology) facilitate coevolutionary adaptation in plant–pre-dispersal seed predator interactions and reveal the mechanisms driving host-related morphological differentiation.

## 1. Introduction

Plant–herbivore interactions are fundamental drivers of evolutionary and ecological dynamics [1]. Insect herbivores commonly exhibit morphological changes in response to heterogeneous environments, such as those imposed by distinct host species. The hypothesis of host-induced morphological plasticity proposes that insects (e.g., pre-dispersal seed predators) can adjust their morphological traits directly in response to specific host characteristics (e.g., nutritional quality [2], physical structure [3]) to improve survival and reproductive success in a given host environment [4]. Understanding host-associated morphological variation in insects may not only reveal the origins of biodiversity and speciation but also offer valuable insights into the ecological dynamics of host plant–insect interactions [5,6].

Host-associated morphological variation (e.g., wing size or shape) is often associated with divergent traits across host plant species [7,8]. Many tephritid flies act as pre-dispersal seed predators, with larvae developing and feeding within host plant flowerheads [9]; adult body size is frequently associated with flowerhead size [10,11,12]. However, few studies have investigated the wing morphological variation in these seed predators across different plant species, especially in natural ecosystems where multiple host species coexist.

In the Zoige area of the Qinghai–Tibet Plateau, *Tephritis* sp. cf. *femoralis* may consist of several host-associated morphological groups that develop in the capitula of various asteraceous plant species. Empirical studies have shown that these fly groups typically produce two or three generations of flies per year [13], and adult females may shift among host plants depending on which plant is flowering when they are ready to lay eggs. Mainly in the second generation, female flies may oviposit into plant capitula, including *Saussurea nigrescens* (SN), *Carduus nutans* (CN), *Leontopodium leontopodioides* (LL) and *Anaphalis lactea* (AL) [9,14]. The eggs hatch into larvae that grow and develop by consuming developing seeds within the capitula. Previous studies have shown that the flowerhead sizes of SN and CN are larger than those of LL and AL, and the emerged fly adults from SN and CN are also larger than those in AL and LL [14]. As fly body size depends on the host plant where females lay eggs [14], the body size of second-generation flies may vary across these different host species. However, the wing size and shape have not been examined across these flowerheads in alpine ecosystems.

Wing geometric morphometrics is a well-established tool for detecting morphological changes [15]. By utilizing wing vein junctions as landmarks, this method provides accurate quantitative data for classifying intraspecific or interspecific variation [16]. Therefore, geometric morphometric analysis of wing variation in alate insects can effectively identify morphological differences among and within species and between sexes [17,18,19].

To quantify wing size and shape variations in *Tephritis* sp. cf. *femoralis* groups between females and males in each plant flowerhead, as well as female and male groups among the four host plants (SN, CN, LL and AL), we performed a geometric morphometric analysis on the wings of these fly groups. Specifically, we hypothesized that (1) the wing size of females is larger than that of males; (2) wing size is larger in larger flowerheads (SN and CN) than in smaller flowerheads (LL and AL); and (3) wing shape variation in both sexes can be distinguished across different host plants.

## 2. Materials and Methods

### 2.1. Study Site

The sampling site is in an alpine meadow in Hongyuan County, Sichuan Province, China, in the eastern Qinghai–Tibet Plateau (32°48′ N, 102°33′ E). The climate is characterized by short, cool summers and long, cold winters. The mean annual air temperature and mean annual precipitation during 1970–2020 were 1.7 °C and 754 mm, respectively (Hongyuan County Meteorological Station, 5.5 km from the study site); over 80% of precipitation occurs from May to September [20].

The study meadow is typically dominated by sedges such as *Kobresia setchwanensis* and *Blysmus sinocompressus*; grasses such as *Agrostis hugoniana*, *Elymus nutans* and *Deschampsia caespitosa*, as well as forb species including *S. nigrescens*, *Potentilla anserina*, *Aster alpinus*, *Gentiana formosa*, *Euphrasia hirtella*, *Trollius farreri*, *Pedicularis* spp. and *Thalictrum alpinum*. Total vegetation coverage exceeds 95%, and the average plant height is approximately 30 cm [21]. The meadow has been under intensive grazing for decades and is now used as summer or winter pasture [22]. Except for cattle grazing, this site currently has no other agricultural use.

The study species *S. nigrescens* flowers from late June to late August, and *A. flavescens* from mid-June to early September, with c. 2 months overlap in flowering time [14]. *Carduus nutans* is a biennial forb that occurs along roadways and other disturbed sites, and it flowers from late May to late August [23]. *Leontopodium leontopodioides* flowers from late May to late September [24].

### 2.2. Tephritid Flies Sampling

Tephritid flies usually lay eggs in the early bud stages of asteraceous plant capitula of flowerheads (a flowerhead may consist of ~35 capitula; see [14]). After the eggs hatch into larvae within the capitula, the larvae typically feed and grow for several days before developing into pupae and then emerging as adults. During the late flower development stage of these four asteraceous plants (late July to September 2023), the capitula were randomly collected. Approximately 300 flowerheads were collected for each plant species. The samplings were then transported to the laboratory, and each flowerhead was individually wrapped in a nylon net bag and hung in a cool, well-ventilated place.

Adults from the four host capitula differ considerably in body size and ovipositor length. They exhibit morphological features typical of the genus *Tephritis*, including two pairs of well-developed frontal setae and two pairs of orbital setae, a distinct ocellus, relatively posterior orbital setae, flattened paravertical and postocular setae, and an entirely black subcostal cell on the reticulated wing. These traits are generic characters of the genus *Tephritis* and do not serve as diagnostic features for any particular species group. Previous DNA barcoding (COI) evidence confirms that flies reared from these host capitula include a lineage closely related to *T. femoralis* [25]. However, definitive species identification remains unfeasible due to the absence of topotypic reference material and host-associated reference sequences for *T. femoralis*. Accordingly, these flies are provisionally designated as four host-associated operational groups: *Tephritis* sp. cf. *femoralis* 1 (Tcf1, from SN), 2 (Tcf2, from CN), 3 (Tcf3, from LL), and 4 (Tcf4, from AL). Voucher specimens of *Tephritis* sp. cf. *femoralis* and the four asteraceous host plants are permanently deposited in the laboratory collection of Pingdingshan University, China, and are available for scientific research upon request.

Because not all capitula may be oviposited by the flies and the fly pupae within the capitula may also be parasitized by the wasps [9], the number of emerging flies was lower than the number of collected capitula. Finally, the numbers of females and males in this study were 40 and 38 for Tcf1, 35 and 34 for Tcf2, 38 and 39 for Tcf3, and 39 and 39 for Tcf4. Once the flies emerged, they were transferred to tubes for further dissection.

### 2.3. Plant Capitula Size

Capitular depth (from the receptacle base to the stigma apex) and diameter (maximum sepal width) were measured for each plant species using a vernier caliper (INSIZE 5610-150; INSIZE, Suzhou, China). Intact capitula from each species, selected for dry biomass measurement, were dried at 65 °C for 48 h, and then weighed to 0.1 mg. The numbers of capitula measured for both depth and diameter were 70 (SN), 66 (CN), 92 (LL) and 79 (AL); the numbers of dry biomass were 70 (SN), 66 (CN), 78 (LL) and 93 (AL).

### 2.4. Geometric Morphometric Analysis

**Figure 1:**
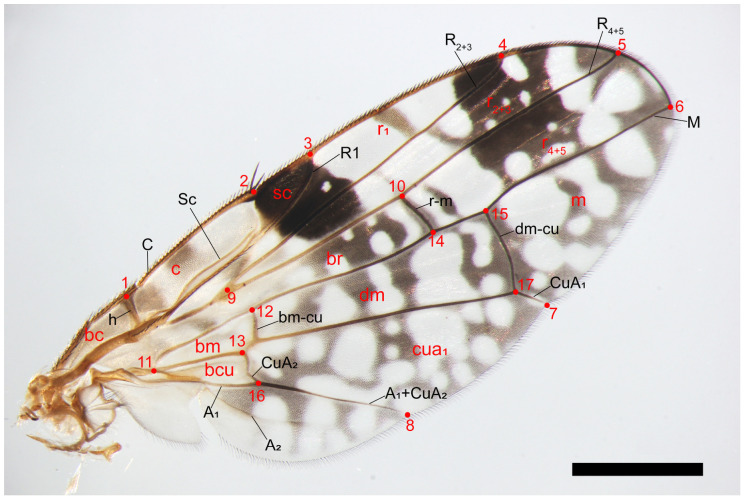
The right wing of a female Tcf1 specimen, showing all 17 landmarks, wing cells, and wing veins. Circular red dots represent landmarks 1–17; red letters label wing cells; black letters with short leader lines pointing to wing veins indicate vein nomenclature. Wing veins: C (costa), Sc (subcosta), R (radius, including R_1_, R_2+3_, R_4+5_), M (media), CuA_1_ (anterior cubitus 1), CuA_2_ (anterior cubitus 2), A_1_+CuA_2_ (first anal vein + anterior cubitus 2), A_1_ (first anal vein), and A_2_ (second anal vein). Wing cells: bc (basal costal cell), c (costal cell), sc (subcostal cell), br (basal radial cell), r_1_ (first radial cell), r_2+3_ (second+third radial cell), r_4+5_ (fourth+fifth radial cell), bm (basal medial cell), bcu (basal cubital cell), dm (discal medial cell), m (medial cell), and cua_1_ (first cubital cell). Scale bar = 1 mm.

The right forewings were cut from the samples under a Nikon SMZ1500 optical microscope (Nikon, Tokyo, Japan). The wing vein terminology of the tephritid fly is shown in Figure 1 and Table 1, based on previous studies [26]. Seventeen homologous landmarks were identified on the forewing (Table 1), and all landmarks are the intersections and endpoints of wing veins, belonging to the type I coordinate points [27]. TPSDig2 software (Version 2.30) was used to digitize the landmarks [28].

Fly wing size was indicated by the wing length and width: the wing length was measured from the axillary region base to landmark 6 of the wing, and the width was defined as the distance between landmarks 3 and 8. Because the aculeus inside the oviscape is not always visible, the ovipositor length was represented by the oviscape length. The female numbers for both wing length and width were 23 (Tcf1), 26 (Tcf2), 26 (Tcf3), and 24 (Tcf4); and for ovipositor length, 24 (Tcf1), 28 (Tcf2), 17 (Tcf3) and 28 (Tcf4). The number of males measured for both wing length and width was 22 (Tcf1), 24 (Tcf2), 27 (Tcf3), and 27 (Tcf4). The female centroid size was 40 (Tcf1), 35 (Tcf2), 38 (Tcf3) and 39 (Tcf4), while the male centroid size was 38 (Tcf1), 34 (Tcf2), 39 (Tcf3) and 39 (Tcf4). The centroid size of each *Tephritis* sp. cf. *femoralis* group for each plant species was calculated using tpsRelw (Version 1.75) [29].

We performed an allometric analysis to evaluate the effect of centroid size on wing shape using multivariate regression in the R package “geomorph” (Version 4.0.7). If significant allometry was detected, wing shape variation was corrected for size effects using regression residuals, and all subsequent statistical analyses and visualizations (PCA, CVA, MANOVA) were based on this size-corrected shape dataset. If no significant allometry was found, uncorrected Procrustes coordinates were retained for all downstream analyses.

All the original coordinates in the TPS file generated by TPSDig2 were converted to Procrustes coordinates using CoordGen8 (Version 6/3/2014). Principal component analysis (PCA) and canonical variate analysis (CVA) were performed using PCAGen8 (Version 4/29/2014) and CVAGen8 (Version 9/30/2014) from the IMP software series [30]. PCA was used to determine the explained percentage of the total variation explained by each principal component (PC). CVA was used to distinguish between groups and identify shape variations associated with canonical variates (CVs). To graphically illustrate variation in wing shape along the resulting PCs and CVs, wireframe outlines of extreme shapes along each axis were created in MorphoJ (Version 1.06d) [31]. A one-factor permutational multivariate analysis of variance (MANOVA) implemented in CVAGen8 was used to test wing shape variation between females and males within each flowerhead species and among female and male groups across the four plant flowerheads, with 999 repeated permutations. A linear discriminant analysis (LDA) was used to calculate the classification accuracy (hit ratio, HR) for the samples using the jack-knife test.

To approach normality, the capitular depth, diameter and dry weight, as well as fly wing length, width and centroid size for each plant species, were all log (*x* + 1)-transformed [32]. The homogeneity of variance was tested using Levene’s test. A significance level of α = 0.05 was used for all statistical tests. One-way ANOVA followed by a Tukey post hoc test (homoscedasticity) or Welch’s ANOVA followed by a Tamhane’s T2 post hoc test (heteroscedasticity) was used to evaluate differences in capitulum diameter, depth, and dry weight, as well as wing size among plant species. A t-test was used to evaluate differences between females and males in each plant species. The ANOVAs and t-tests were conducted in R 4.4.2 [33].

## 3. Results

### 3.1. Size of Capitula

**Figure 2:**
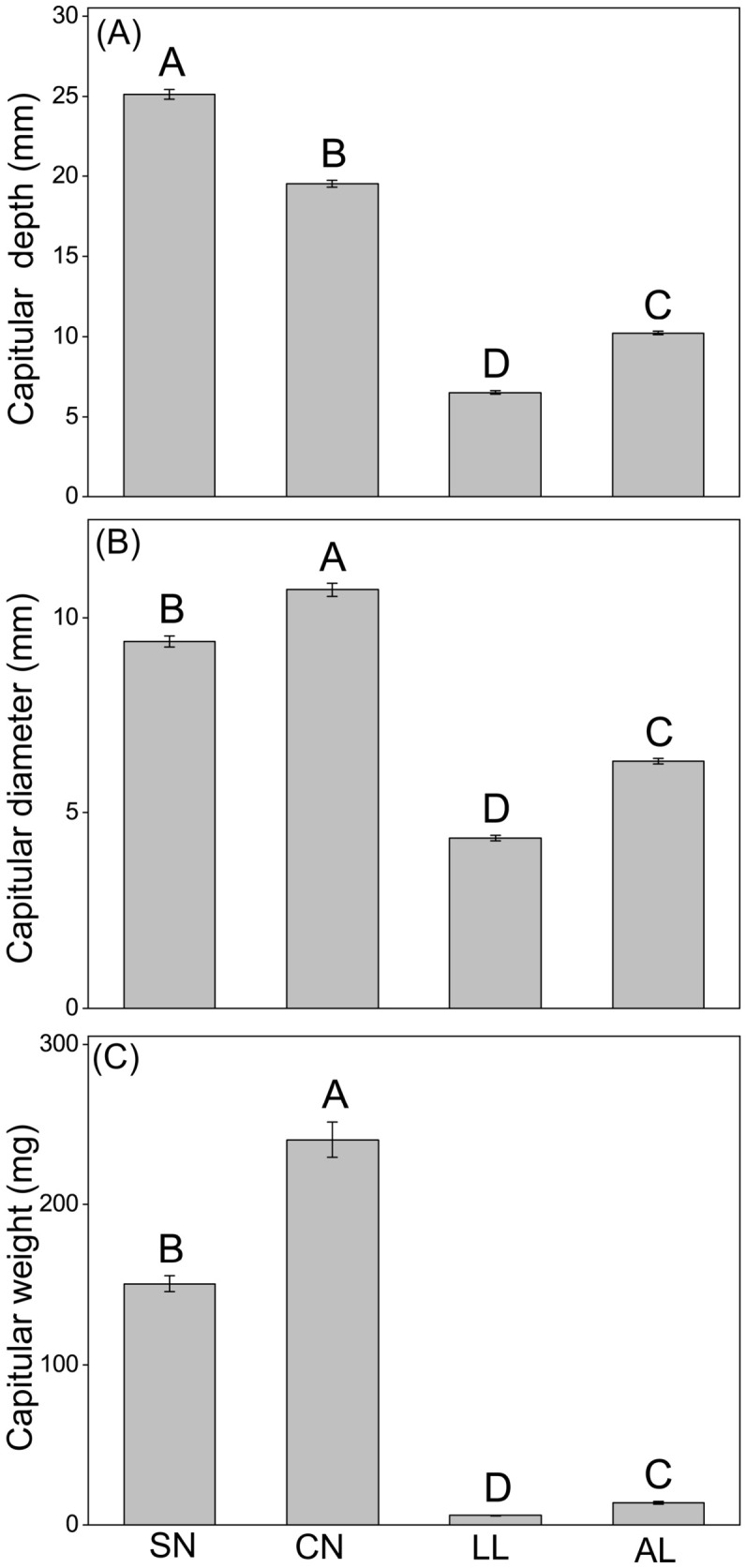
Variations in plant flowerhead capitulum depth (**A**), capitulum diameter (**B**) and capitulum dry weight (**C**) of SN, CN, LL and AL. Different uppercase letters above the error bars denote statistically significant differences at *p* < 0.05 level among SN, CN, LL and AL.

Significant differences were found in the capitular depth (Welch’s ANOVA: *F* = 2110.59, *p* < 0.001), diameter (Welch’s ANOVA: *F* = 778.32, *p* < 0.001) and dry weight (Welch’s ANOVA: *F* = 2710.48, *p* < 0.001) among these four host species. The capitular diameter and dry weight of SN were significantly smaller than those of CN (Figure 2B,C), while the depth of SN was larger than that of CN (Figure 2A). The capitular depth, diameter, and dry weight of both SN and CN were significantly greater than those of LL and AL (Figure 2). The capitular depth, diameter and dry weight of AL were all significantly larger than those of LL (Figure 2).

### 3.2. Differences in Wing Size Between Females and Males and Female Ovipositor Length

**Figure 3:**
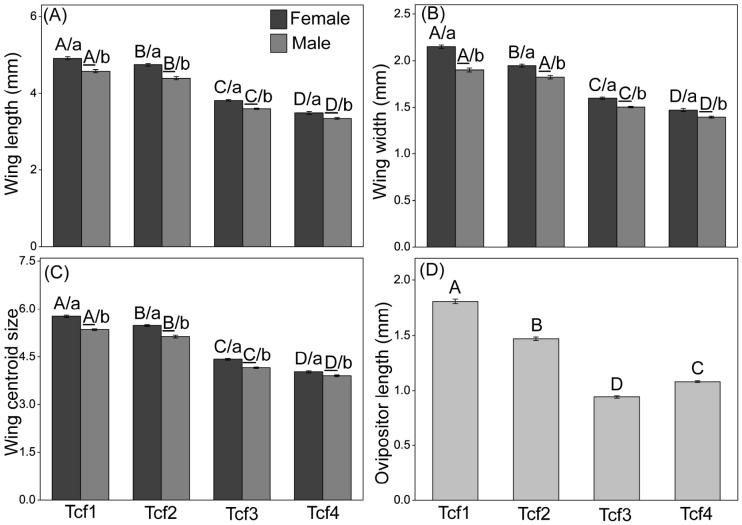
The variations in wing length (**A**), wing width (**B**), wing centroid size (**C**), and ovipositor length (**D**) of *Tephritis* sp. cf. *femoralis* 1, 2, 3, and 4 from different host plant flowerheads of SN, CN, LL, and AL, respectively. Different uppercase letters above the error bars denote statistically significant differences at the *p* < 0.05 level among female groups of Tcf1, Tcf2, Tcf3, and Tcf4, and underlined uppercase letters for male groups of Tcf1, Tcf2, Tcf3, and Tcf4. Different lowercase letters denote statistically significant differences between males and females within Tcf1, Tcf2, Tcf3, and Tcf4.

All female wing lengths, widths and centroid sizes were larger than those of males from each host plant flowerhead (Figure 3A–C). There were significant differences among female groups in wing length (one-way ANOVA: *F*_3, 95_ = 434.12, *p* < 0.001), width (one-way ANOVA: *F*_3, 95_ = 305.1, *p* < 0.001) and centroid size (one-way ANOVA: *F*_3, 148_ = 661.92, *p* < 0.001), as well as in male wing length (one-way ANOVA: *F*_3, 95_ = 352.07, *p* < 0.001), width (one-way ANOVA: *F*_3, 95_ = 301.36, *p* < 0.001) and centroid size (one-way ANOVA: *F*_3, 146_ = 638.16, *p* < 0.001) among the four plant flowerheads. The order of wing size in female groups (from largest to smallest, the same below) among these flowerheads was Tcf1 (SN), Tcf2 (CN), Tcf3 (LL), Tcf4 (AL), and this is also the case for the male group (except that male wing width showed no difference between Tcf1 and Tcf2) (Figure 3A–C). Significant differences in female ovipositor length existed among these flowerheads (one-way ANOVA: *F*_3, 93_ = 752.55, *p* < 0.001), and the order of ovipositor length was Tcf1 (SN), Tcf2 (CN), Tcf4 (AL), and Tcf3 (LL) (Figure 3D).

### 3.3. Allometric Analysis Between Wing Shape and Centroid Size

Significant allometry was detected only in males of Tcf3 (*p* = 0.019 < 0.05), while the other groups were all non-significant (females of Tcf1: *p* = 0.18; females of Tcf2: *p* = 0.22; females of Tcf3: *p* = 0.09; females of Tcf4: *p* = 0.34; males of Tcf1: *p* = 0.79; males of Tcf2: *p* = 0.42; males of Tcf4: *p* = 0.37).

### 3.4. Wing Shape Variation Between Females and Males in Each Flowerhead

**Figure 4:**
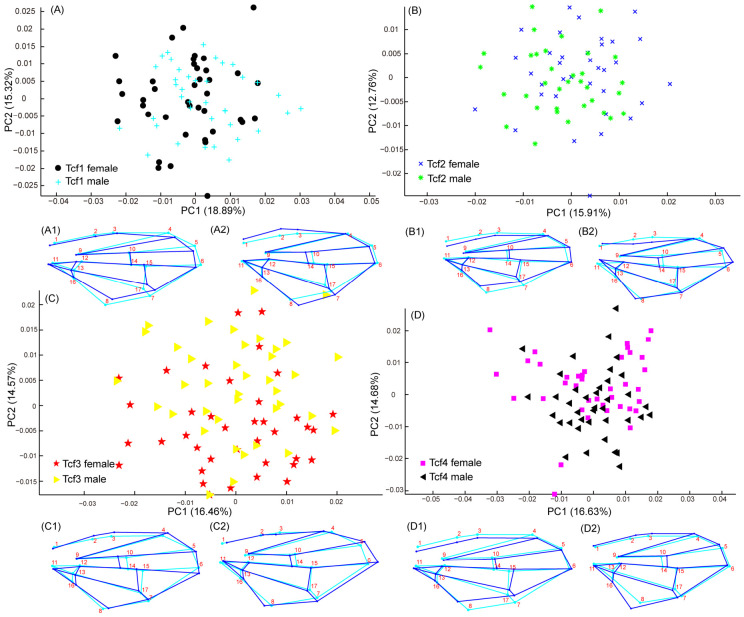
PCA scatter plots of the shape variation based on female and male forewings of *Tephritis* sp. cf. *femoralis* from SN (**A**), CN (**B**), LL (**C**), and AL (**D**). Shape changes associated with PC1 (**A1**) and PC2 (**A2**) in SN, PC1 (**B1**) and PC2 (**B2**) in CN, PC1 (**C1**) and PC2 (**C2**) in LL and PC1 (**D1**) and PC2 (**D2**) in AL are shown. A dark blue wireframe graph (solid dots) is compared with the overall mean shape (open bright blue dots). Red numbers on the wireframes correspond to the 17 landmarks defined in Figure 1.

In PCA, each PC explained progressively less variance, with PC1 (15~20%) and PC2 (14~16%) of shape variation between female and male in Tcf1, Tcf2, Tcf3, and Tcf4 (Figure 4). All female specimens from each flowerhead were clustered together with male specimens in the PCA. Shape changes associated with PC1 represented a constriction of anal area (landmarks 7 and 8) in Tcf1–Tcf4 (Figure 4(A1–D1)); distal part (landmarks 5 and 6) in Tcf1 (Figure 4(A1)), costal margin (landmarks 1, 2, and 3) in Tcf1, Tcf2 and Tcf4 (Figure 4(A1,B1,D1)); and crossveins of r-m (between landmarks 10 and 14) and dm-cu (between landmarks 15 and 17) in Tcf3 (Figure 4(C1)); as well as an elongation of distal part (landmarks 5 and 6) in Tcf2 (Figure 4(B1)). PC2 represented a constriction of the distal part (landmarks 5 and 6) in Tcf1 and Tcf4 (Figure 4(A2,D2)), and costal margin (landmarks 2 and 3) in Tcf2 and Tcf3 (Figure 4(B2,C2)); it aslo represented an elongation of anal area (landmarks 7 and 8) in Tcf1, Tcf2, Tcf3, and Tcf4 (Figure 4(A2–D2)), and distal part (landmarks 5 and 6) in Tcf2 and Tcf3 (Figure 4(B2,C2)).

**Figure 5:**
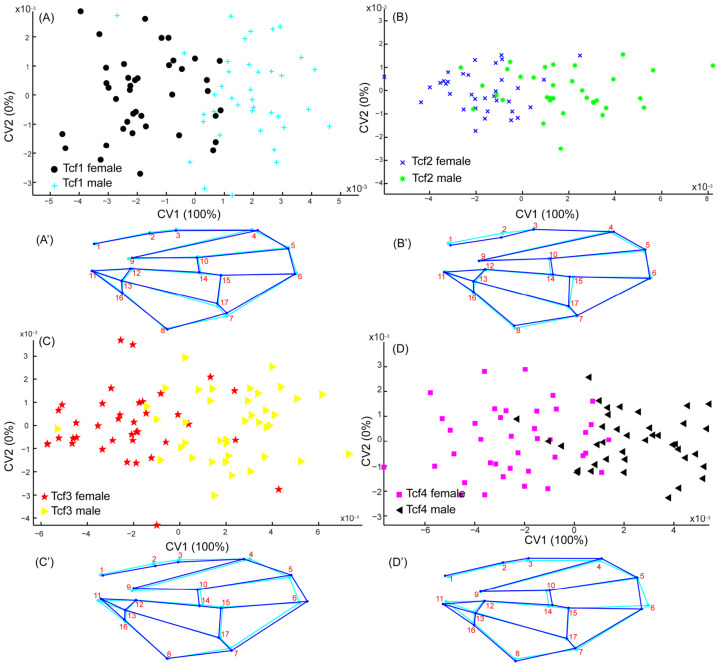
CVA scatter plots of the shape variation between female and male forewings of *Tephritis* sp. cf. *femoralis* from SN (**A**), CN (**B**), LL (**C**), and AL (**D**). Shape changes associated with CV1 (**A’**) in SN, CV1 (**B’**) in CN, CV1 (**C’**) in LL and CV1 (**D’**) in AL are shown. A dark blue wireframe graph (solid dots) is compared with the overall mean shape (open bright blue dots). Red numbers on the wireframes correspond to the 17 landmarks defined in Figure 1.

Significant shape differences between female and male wings were found in Tcf1 (MANOVA: *F*_1, 76_ = 2.01, *p* = 0.035), Tcf3 (MANOVA: *F*_1, 75_ = 2.47, *p* = 0.005), and Tcf4 (MANOVA: *F*_1, 76_ = 2.82, *p* = 0.003), while no significant difference was observed in Tcf2 (MANOVA: *F*_1, 67_ = 1.55, *p* = 0.078). Female specimens could be separated from male specimens, with partial overlap in these flowerheads. Shape changes associated with CV1 represented a constriction of the costal margin (landmarks 1, 2, and 3) in Tcf2 and Tcf3 (Figure 5(B’,C’)) and the distal part (landmark 6) in Tcf4 (Figure 5(D’)), as well as an expansion of the distal part (landmark 6) in Tcf3 (Figure 5(C’)).

### 3.5. Wing Shape Variation in Female and Male Groups Across Four Flowerheads

**Figure 6:**
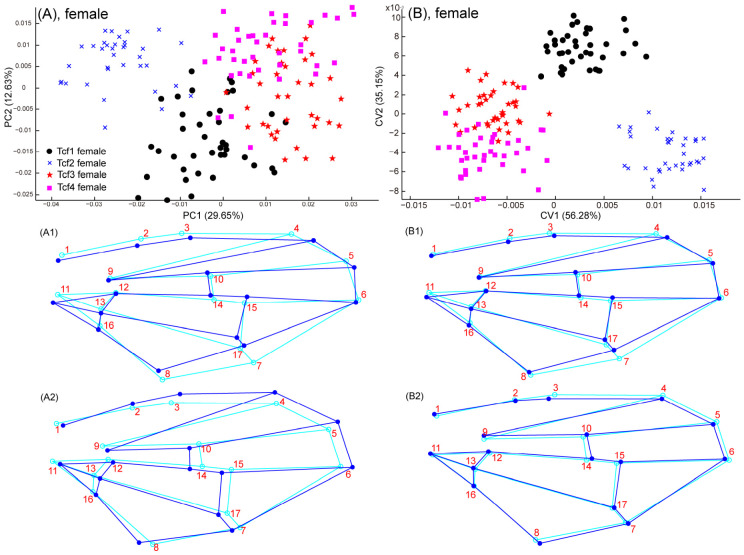
PCA scatter plots (**A**) and CVA scatter plots (**B**) of the shape variation based on female forewings of *Tephritis* sp. cf. *femoralis* from different flowerheads. Wireframe graphs illustrate the shape variation associated with PC1 (**A1**), PC2 (**A2**), CV1 (**B1**) and CV2 (**B2**). A dark blue wireframe graph (solid dots) is compared with the overall mean shape (open bright blue dots). Red numbers on the wireframes correspond to the 17 landmarks defined in Figure 1.

**Figure 7:**
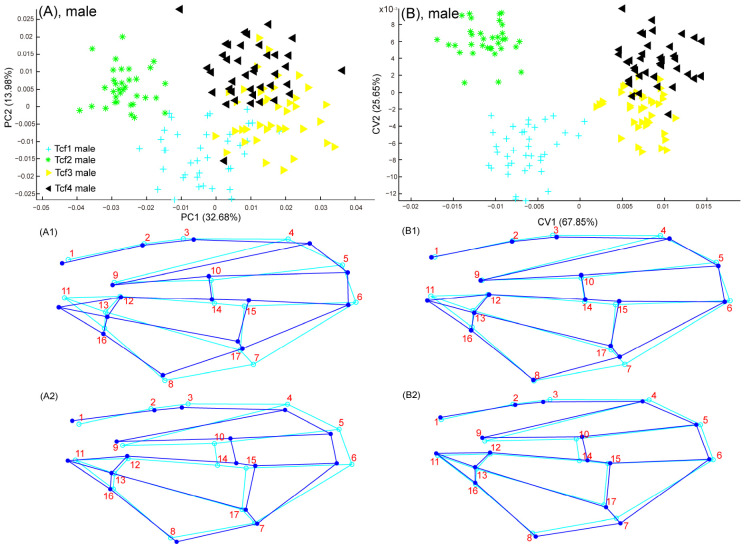
PCA scatter plots (**A**) and CVA scatter plots (**B**) of the shape variation based on male forewings of *Tephritis sp.* cf. *femoralis* from different flowerheads. Wireframe graphs illustrate the shape variations associated with PC1 (**A1**), PC2 (**A2**), CV1 (**B1**) and CV2 (**B2**). A dark blue wireframe graph (solid dots) is compared with the overall mean shape (open bright blue dots). Red numbers on the wireframes correspond to the 17 landmarks defined in Figure 1.

In PCA, the first two PCs accounted for 42.28% of total shape variation in females (Figure 6A), and 46.66% in males (Figure 7A). In females, PC1 represented a constriction of costal margin (landmarks 1, 2, 3, and 4) and anal area (landmarks 7 and 8), and an expansion of the wing proximal part (landmark 11) (Figure 6(A1)); PC2 represented a constriction of crossveins of r-m and dm-cu, and an expansion of costal margin and distal part (Figure 6(A2)). In males, PC1 represented a constriction of costal margin (landmarks 1 and 2) and anal area (landmarks 7 and 8), and an expansion of the wing proximal part (landmark 11) (Figure 7(A1)); PC2 represented a constriction of the costal margin (landmarks 2, 3 and 4) and distal part (landmarks 5 and 6), and an elongation of crossveins of r-m and dm-cu (Figure 7(A2)).

MANOVA showed that there were significant variations in wing shape in both female groups (*F*_3, 148_ = 24.34, *p* = 0.001) and male groups (*F*_3, 146_ = 27.96, *p* = 0.001) across these flowerheads. CV1 (56.28%, Wilks’ λ = 0.0027, *p* < 0.001) and CV2 (35.13%, Wilks’ λ = 0.069, *p* < 0.001) accounted for 91.43% in females, while CV1 (67.85%, Wilks’ λ = 0.0077, *p* < 0.001) and CV2 (25.65%, Wilks’ λ = 0.0934, *p* < 0.001) accounted for 93.5% in males of the total shape variation. Canonical variable analysis of both female and male groups showed that Tcf1 and Tcf2 could be separated from Tcf3 and Tcf4, whereas Tcf3 and Tcf4 partially overlapped (Figure 6B and Figure 7B). In females, shape changes associated with CV1 explained the most discrimination among host plants, representing a constriction of costal margin (landmarks 1, 2, and 3) and anal area (landmarks 7 and 8), and an expansion of the wing proximal part (landmark 11) (Figure 6(B1)), and CV2 represented a constriction of the distal part (landmarks 5 and 6) and an expansion of anal area (landmarks 7 and 8), and crossveins of the r-m and dm-cu (Figure 6(B2)). In males, shape changes associated with CV1 also explained the most discrimination among host plants, representing a constriction of anal area (landmark 7) and an expansion of the proximal part of the wing (landmark 11) (Figure 7(B1)), and CV2 represented a constriction of the distal part (landmarks 5 and 6), and an expansion of anal area (landmarks 7 and 8), and crossveins of the r-m and dm-cu (Figure 7(B2)).

LDA achieved 92.11% correct classification for females and 92.00% for males, indicating strong discrimination among the four host-associated groups. For females, the hit ratio for assignment to the correct group was 97.5% (39 of 40) for Tcf1, 100% (35 of 35) for Tcf2, 84.21% (32 of 38) for Tcf3 and 87.18% (34 of 39) for Tcf4. For males, the hit ratio for assignment to the correct group was 97.37% (37 of 38) for Tcf1, 100% (34 of 34) for Tcf2, 92.31% (36 of 39) for Tcf3 and 79.49% (31 of 39) for Tcf4.

## 4. Discussion

The present study showed that the wing size of females was larger than that of males in each plant flowerhead, and the wing size of the flies developing in larger flowerheads such as *S. nigrescens* and *C. nutans* was larger than that in *L. leontopodioides* and *A. lactea*, consistent with previous studies [34]. Wing shape between females and males could be distinguished in Tcf1, Tcf3 and Tcf4. Moreover, both the female and male groups showed a similar pattern of wing morphological variation among these flowerheads, and both female and male groups across these flowerheads can be separated. Our study presents a new case of wing shape variation in tephritid flies in alpine asteraceous flowerhead–pre-dispersal seed predator systems.

Overall, allometric effects on wing shape were weak and negligible in the present study. Multivariate regression revealed significant allometry between wing shape and centroid size in males of Tcf3 (from *Leontopodium leontopodioides*), whereas no significant allometry was detected in any other female or male groups. These results indicate that size-related allometric effects had little overall influence on wing shape variation and that the observed host-associated wing shape divergence was primarily driven by host plant traits rather than allometric scaling, thereby supporting the robustness of our morphological comparisons.

Sexual morphological differences are prevalent in many insect taxa and are a major source of phenotypic variation [35,36,37]. The ecological consequences of variation in external wing morphology between females and males often reflect adaptive evolutionary relationships with flight performance and behavioral activities, as reported in Diptera and Hymenoptera [38,39,40,41]. In this study, females exhibited significantly larger wings than males, a phenomenon known as female-biased size dimorphism. This pattern is also consistent with previous studies [23], confirming that the wing length may serve as a surrogate measure of body size in Tephritidae [42,43]. Ecologically, large wings and body size enable females to support a heavier abdomen loaded with eggs while searching for suitable oviposition sites [44,45]. Moreover, larger females may produce more and larger eggs in more flowerheads, thereby enhancing offspring survival, especially at high altitudes with a short growing season [46]. On the contrary, the small size of males was considered to be associated with early maturation, because rapid male development can increase mating success and give males more opportunities to occupy better territories [47].

Although wing shape differed between females and males in SN, LL, and AL, individuals of both sexes clustered closely in the PCA and showed only partial separation in CVA, similar to previous studies [48]. These results suggest that wing shape variation may be less effective than wing size for discriminating sexes within the same host-associated group. Sexual differences in wing venation are closely linked to wing size variation; e.g., R_4+5_ (landmark 5) and M (landmark 6) correlate with wing length, and A_1_+CuA_2_ (landmark 8) is associated with wing width [49]. Thus, the observed wing shape differences between females and males likely arise from adaptive body size adjustments in response to flowerhead size variation during long-term coevolution between flies and their host plants.

Host plant traits are closely correlated with insect morphological differentiation [50,51,52]. Although the capitulum size (depth, diameter and dry weight) was not fully consistent with the fly wing size (length, width, and centroid size) among the four flowerheads, the capitular depth was strongly matched with female ovipositor length, in line with previous studies [12]. Flies usually lay eggs into the capitulum directly above the stigma [53]; therefore, the ovipositor length must be sufficient to reach the oviposition site, making it necessarily matched with the capitular depth. This directly reflects the adaptive matching between the insect’s reproductive structure and the host’s physical traits. It is likely that greater capitulum depth would support larger pupae in terms of physical space, as pupae usually orient vertically within fully opened flowerheads [54], thus possibly resulting in larger wing sizes in SN and CN than those in LL and AL. These findings also suggest that ovipositor length contributes to the discrimination of female flies associated with different host plant flowerheads.

In the present study, shape variation along the first axis of both the four female and four male groups across the four plants was characterized by a constriction of costal margin, distal part and anal angle area, corresponding to a smaller wing size in flies from smaller flowerheads. The most plausible explanation is that host resources constrain adult body size; that is, SN and CN have larger flowerheads (here indicated by the flowerhead diameter, depth, and dry weight) and hence provide greater nutrient contents than LL and AL [14]. Similar positive relationships between host plant size and body size of endophagous larvae were also documented in previous studies on tephritid flies [55,56].

The pattern of adult oviposition across different flowerheads may also be attributed to plant phenology. Previous studies have shown that the flies in the second generation primarily oviposit into the dominant species *S. nigrescens* (flowers from late June to late August), *A. flavescens* and *A. lactea* (from mid-June to early September) [14]. Although *A. flavescens* and *A. lactea* are regarded as suboptimal oviposition hosts [9], preferred hosts such as *S. nigrescens* are often limited in availability. Consequently, high densities of *Tephritis* sp. cf. *femoralis* females are forced to oviposit on these less suitable plant species, which have higher flowerhead densities and longer flowering periods [9,57]. This pattern is consistent with previous studies indicating that fly populations are closely linked to host plant phenology [11,58].

In addition to capitulum size and phenology, host origin (native vs. exotic) represents another important driver of host-associated morphological divergence in *Tephritis* sp. cf. *femoralis. Carduus nutans* is an exotic species [59], mainly distributed along roadsides, whereas the other three species (*S. nigrescens*, *L. leontopodioides*, *A. lactea*) are endemic to the local community. Exotic hosts often create novel ecological niches with distinct microenvironmental conditions and nutritional profiles compared with coevolved native hosts [7,60]. Notably, the deep phylogenetic distance between *C. nutans* (Carduoideae: Cardueae) and the three native hosts (Asteroideae: Gnaphalieae) likely contributes to the morphological differentiation observed in Tcf2 relative to the other groups. Consistent with findings in other tephritid flies [7,61] and aphids [60], our results indicate that individuals developing in the exotic host CN exhibited significant wing size and shape differentiation relative to those from native hosts. This reinforces the idea that exotic host plants can accelerate host-associated morphological divergence in pre-dispersal seed predators and extends our understanding of how both native and exotic host plants jointly shape morphological variation in *Tephritis* sp. cf. *femoralis* groups in alpine plant–insect interactions.

Wing shape variation exhibited consistent patterns between female and male groups across the plant flowerheads. Both female and male groups could be distinguished across four plant flowerheads, indicating that host-associated variation in wing shape is independent of sex. Except for the wing veins (R_4+5_, M and A_1_+CuA_2_) associated with the wing length and width as aforementioned, r-m and R_2+3_ (landmark 4) are the main wing veins that form the R_2+3_ cell, which are the main areas of aerodynamic force production [62]. Thus, variation in these wing veins may contribute to flight performance in flies associated with different flowerheads. It is worth noting that the fly specimens from LL and AL showed partial overlap in both the female and male groups in PCA and CVA, resulting in relatively low classification accuracy (hit ratio) for these two groups. Given that greater morphological divergence typically arises from greater divergence in host traits [63,64,65], we speculate that the smaller relative difference in capitulum depth between AL and LL (i.e., (AL depth − LL depth)/LL depth = 57%) compared to that between SN and LL (286%), and between CN and LL (200%) accounts for the higher morphological similarity and overlapping wing shapes.

Tephritid flies occupying different ecological niches may have additional ecological consequences. First, although different generations of flies developing in different flowerheads may alleviate intense intraspecific or interspecific competition, the interactions within a single capitulum are primarily driven by host constraints and maternal strategies rather than competitive exclusion. Empirical studies have shown that typically only one fly individual emerges from a single flowerhead capitulum, even when multiple insect species oviposit into the same capitulum [53,66]. This pattern likely depends on the host plant’s capitulum size, reflecting a female reproductive strategy of matching oviposition to available biomass, as well as potential parasitoid pressure [9], rather than solely larval competition [66]. Second, flies utilizing the suboptimal host exert significant effects on the population dynamics and species diversity of their associated parasitoids [9]. Moreover, flies with larger body sizes in SN-dominated plants may exert stronger feeding pressure on common plant species than on rare ones, resulting in negative density dependence and thereby contributing to species coexistence [53].

## 5. Conclusions

In summary, four morphological groups of *Tephritis* sp. cf. *femoralis* (1, 2, 3, 4) are associated with the traits of their hosts. Specifically, the wing size of females is larger than that of males within each host plant. The wing size of flies emerging from the larger capitula (depth, diameter, and dry mass) of *Saussurea nigrescens* and *Carduus nutans* is larger than that of *Tephritis* sp. cf. *femoralis* groups from the smaller capitula of *Leontopodium leontopodioides* and *Anaphalis lactea*. Meanwhile, the female ovipositor length showed a tight adaptive match with host capitulum depth, highlighting a key reproductive trait shaped by host structural constraints. Moreover, the wing shape of both the female and male groups across these flowerheads can be distinguished. LDA provided a high correct classification rate for female and male groups. Collectively, although four operational groups were defined, our morphological analyses suggest they may represent three distinct morphospecies (with potential overlap between Tcf3 and Tcf4) with unresolved taxonomic status. Definitive identification requires comparison with topotypic material of *T. femoralis* and integrative taxonomic analysis combining molecular and morphological evidence.

## Figures and Tables

**Table 1:** The number and description of the 17 anatomical landmarks used to characterize wing shape in *Tephritis* sp. cf. *femoralis*.

Landmark Number	Landmark Description
1	Intersection of humeral (h) and costal veins (C)
2	Intersection of subcostal vein (Sc) with margin
3	Intersection of vein R_1_ with margin
4	Intersection of veins R_2+3_ with margin
5	Intersection of veins R_4+5_ with margin
6	Intersection of vein M with apical margin
7	Intersection of vein CuA_1_ with apical margin
8	Intersection of vein A_1_+CuA_2_ with posterior margin
9	Intersection of vein R_2+3_ and R_4+5_
10	Intersection of vein r-m and R_4+5_
11	Intersection of vein M and base of cell bm
12	Intersection of veins M and bm-cu
13	Intersection of veins CuA_1_ and CuA_2_
14	Intersection of veins M and r-m
15	Intersection of veins M and dm-cu
16	Intersection of vein A_1_ with apex of cell bcu
17	Intersection of veins CuA_1_ and dm-cu

## Data Availability

The original contributions presented in this study are included in the article. Further inquiries can be directed to the corresponding author.

## Abbreviations

The following abbreviations are used in this manuscript:

Tcf1	*Tephritis* sp. cf. *femoralis* 1 *(Saussurea nigrescens)*
Tcf2	*Tephritis* sp. cf. *femoralis* 2 *(Carduus nutans)*
Tcf3	*Tephritis* sp. cf. *femoralis* 3 *(Leontopodium leontopodioides)*
Tcf4	*Tephritis* sp. cf. *femoralis* 4 *(Anaphalis lactea)*
SN	*Saussurea nigrescens*
CN	*Carduus nutans*
LL	*Leontopodium leontopodioides*
AL	*Anaphalis lactea*
